# Supplementary material for: Portable smartphone-integrated paper sensors for fluorescence detection of As(III) in groundwater
Source: R Soc Open Sci. 2020 Dec 16;7(12):201500. doi: 10.1098/rsos.201500 (PMC7813225; doi:10.1098/rsos.201500)
Supplement: Supporting Information for Portable smartphone integrated paper sensors for fluorescence detection of As(III) in groundwater [file rsos201500supp1.docx]

**Supporting Information**

**Portable smartphone integrated paper sensors for fluorescence detection of As(III) in groundwater**

Sha Liu, Yong Li*, Chao Yang, Liqiang Lu, Yulun Nie, Xike Tian

Faculty of Materials Science and Chemistry, China University of Geosciences, Wuhan 430074, China

*Corresponding author. Tel.: +86 027 67883739; Email address: liyong07@126.com

**Figure and table captions:**

**Materials and instrumentation**

**Figure S1** Fluorescence intensities of Cu nanoclusters when the incubation time varying from 10 min to 60 min was employed.

**Figure S2** Fluorescence intensities of Cu nanoclusters when the incubation temperatures varying from 25℃ to 85℃ were employed.

**Figure S3** Fluorescence intensities of Cu nanoclusters when the concentrations of glutathione varying from 10 mg/mL to 70 mg/mL were employed.

**Figure S4** Fluorescence intensities of Cu nanoclusters when pH values varying from 4 to 10 were employed.

**Figure S5** Fluorescence intensities of Cu nanoclusters when different copper precursors were employed.

**Figure S6** Fluorescence excitation and emission spectra of Cu nanoclusters. Inset: Images of Cu nanoclusters powder and solution under sunlight and UV light.

**Figure S7** Fluorescence emission spectra of Cu nanoclusters, glutathione and Cu(NO_3_)_2_.

**Figure S8** Size distribution of Cu nanoclusters and the size is about 3 nm.

**Figure S9** Fluorescence spectra of Cu nanoclusters in the presence and absence of As(III). *Inset*: Fluorescence images of Cu nanoclusters in the presence (A) and absence (B) of As(III) under a 365 nm UV light.

**Figure S10** The influence of pH values varying from 4 to 10 on the sensitive detection of As(III) by Cu nanoclusters.

**Figure S11** The selectivity of Cu nanoclusters toward As(III) over common cations. The black bars represent the fluorescence response of Cu nanoclusters toward As(III) (0.5 μM) and other interfering cations (As(III) (10 μM). The red bars represent the fluorescence response Cu nanoclusters toward interfering cations (10 μM) in the presence of 0.5 μM As(III). All experiments were carried out in BR buffer solution.

**Table S1** Comparison of detection limit of Cu nanoclusters with other previously reported sensors for As(III) detection.

**Table S2** The recovery test of As(III) in ultrapure water, tap water and groundwater samples by Cu nanoclusters ^a^.

**Table S3** As(III) concentrations determination from HPLC-ICP-MS and smartphone integrated paper sensors.

**Materials and instrumentation**

Copper nitrate, glutathione, phosphoric acid, glacial acetic acid, boric acid were purchased from [Sinopharm](http://cn.bing.com/dict/clientsearch?mkt=zh-CN&setLang=zh&form=BDVEHC&ClientVer=BDDTV3.5.0.4311&q=%E5%9B%BD%E8%8D%AF%E9%9B%86%E5%9B%A2%E5%8C%96%E5%AD%A6%E8%AF%95%E5%89%82%E6%9C%89%E9%99%90%E5%85%AC%E5%8F%B8) [Chemical](http://cn.bing.com/dict/clientsearch?mkt=zh-CN&setLang=zh&form=BDVEHC&ClientVer=BDDTV3.5.0.4311&q=%E5%9B%BD%E8%8D%AF%E9%9B%86%E5%9B%A2%E5%8C%96%E5%AD%A6%E8%AF%95%E5%89%82%E6%9C%89%E9%99%90%E5%85%AC%E5%8F%B8) [Reagent](http://cn.bing.com/dict/clientsearch?mkt=zh-CN&setLang=zh&form=BDVEHC&ClientVer=BDDTV3.5.0.4311&q=%E5%9B%BD%E8%8D%AF%E9%9B%86%E5%9B%A2%E5%8C%96%E5%AD%A6%E8%AF%95%E5%89%82%E6%9C%89%E9%99%90%E5%85%AC%E5%8F%B8) [Co](http://cn.bing.com/dict/clientsearch?mkt=zh-CN&setLang=zh&form=BDVEHC&ClientVer=BDDTV3.5.0.4311&q=%E5%9B%BD%E8%8D%AF%E9%9B%86%E5%9B%A2%E5%8C%96%E5%AD%A6%E8%AF%95%E5%89%82%E6%9C%89%E9%99%90%E5%85%AC%E5%8F%B8). [Ltd](http://cn.bing.com/dict/clientsearch?mkt=zh-CN&setLang=zh&form=BDVEHC&ClientVer=BDDTV3.5.0.4311&q=%E5%9B%BD%E8%8D%AF%E9%9B%86%E5%9B%A2%E5%8C%96%E5%AD%A6%E8%AF%95%E5%89%82%E6%9C%89%E9%99%90%E5%85%AC%E5%8F%B8). The interfering anions NO_3_^-^, CO_3_^2-^, HCO_3_^-^, SO_4_^2-^, C_2_O_4_^2-^, PO_4_^3-^, BrO_3_^-^, ClO_4_^-^, F^-^, Cl^-^, Br^-^, I^-^ with sodium ion as counter cations, and interfering cation Na^+^, K^+^, Al^3+^, Fe^3+^, Fe^2+^, Cu^2+^, Zn^2+^, Cd^2+^, Hg^2+^, Ca^2+^, Mg^2+^ and Pb^2+^ with nitrate as counter anions were also obtained from [Sinopharm](http://cn.bing.com/dict/clientsearch?mkt=zh-CN&setLang=zh&form=BDVEHC&ClientVer=BDDTV3.5.0.4311&q=%E5%9B%BD%E8%8D%AF%E9%9B%86%E5%9B%A2%E5%8C%96%E5%AD%A6%E8%AF%95%E5%89%82%E6%9C%89%E9%99%90%E5%85%AC%E5%8F%B8) [Chemical](http://cn.bing.com/dict/clientsearch?mkt=zh-CN&setLang=zh&form=BDVEHC&ClientVer=BDDTV3.5.0.4311&q=%E5%9B%BD%E8%8D%AF%E9%9B%86%E5%9B%A2%E5%8C%96%E5%AD%A6%E8%AF%95%E5%89%82%E6%9C%89%E9%99%90%E5%85%AC%E5%8F%B8) [Reagent](http://cn.bing.com/dict/clientsearch?mkt=zh-CN&setLang=zh&form=BDVEHC&ClientVer=BDDTV3.5.0.4311&q=%E5%9B%BD%E8%8D%AF%E9%9B%86%E5%9B%A2%E5%8C%96%E5%AD%A6%E8%AF%95%E5%89%82%E6%9C%89%E9%99%90%E5%85%AC%E5%8F%B8) [Co](http://cn.bing.com/dict/clientsearch?mkt=zh-CN&setLang=zh&form=BDVEHC&ClientVer=BDDTV3.5.0.4311&q=%E5%9B%BD%E8%8D%AF%E9%9B%86%E5%9B%A2%E5%8C%96%E5%AD%A6%E8%AF%95%E5%89%82%E6%9C%89%E9%99%90%E5%85%AC%E5%8F%B8). [Ltd](http://cn.bing.com/dict/clientsearch?mkt=zh-CN&setLang=zh&form=BDVEHC&ClientVer=BDDTV3.5.0.4311&q=%E5%9B%BD%E8%8D%AF%E9%9B%86%E5%9B%A2%E5%8C%96%E5%AD%A6%E8%AF%95%E5%89%82%E6%9C%89%E9%99%90%E5%85%AC%E5%8F%B8). Ultrapure water (18.25 MΩ·cm^-1^) was obtained from a Millipore water purification system and used throughout the experiments. Britton-Robinson (BR) buffer solution was prepared by mixing 0.04mol·L^-1^ phosphoric acid, acetic acid and boric acid, then adjusted using 0.2mol·L^-1^ NaOH.

All fluorescence measurements were performed on a Hitachi F-7000 fluorescence spectrophotometer (Tokyo, Japan). Ultraviolet–visible (UV–Vis) spectra were measured on a Perkin Elmer Lambda 35 spectrophotometer. Fourier transform infrared (FT IR) spectra were collected with an EQUNOX 55 instrument using the diffuse reflectance scanning disc technique from 4000 cm^−1^ to 400 cm^−1^ at room temperature. Transmission electron microscopy (TEM) was performed with a JEOL 2000EX (JEOL, Japan) operated at 200 kV. X-ray photoelectron spectroscopy (XPS) was performed with a MULTILAB2000 electron spectrometer from VG Scientific using 300 W Al Kα radiations. Particle size distribution was recorded by a Zetasizer Nano ZS90. An ELAN DRC-e High performance liquid chromatography coupled with inductively coupled plasma mass spectrometry (HPLC-ICP-MS) (PerkinElmer, USA) system was used for As(III) concentration determination.


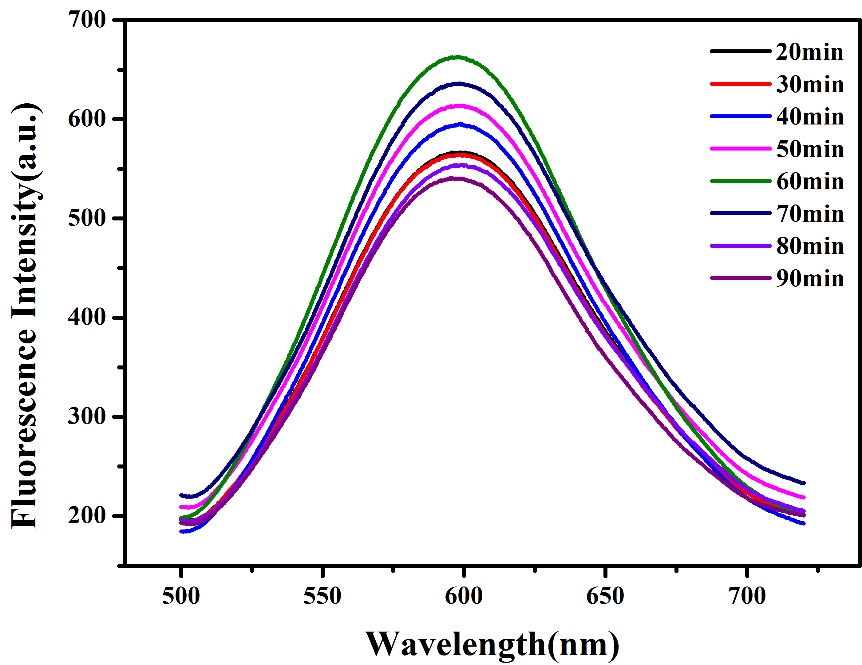


**Figure S1** Fluorescence intensities of Cu nanoclusters when the incubation time varying from 10 min to 60 min was employed.


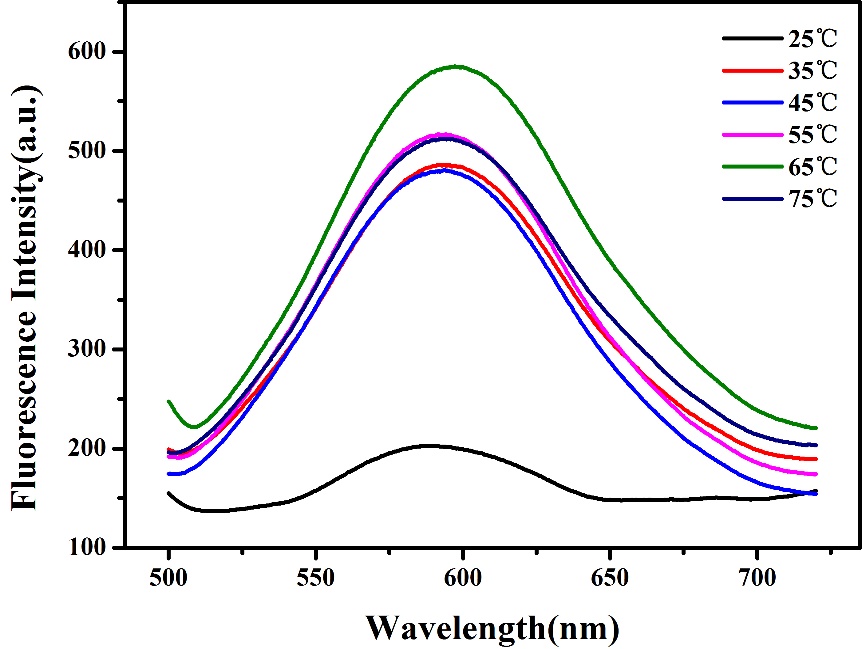


**Figure S2** Fluorescence intensities of Cu nanoclusters when the incubation temperatures varying from 25℃ to 85℃ were employed.


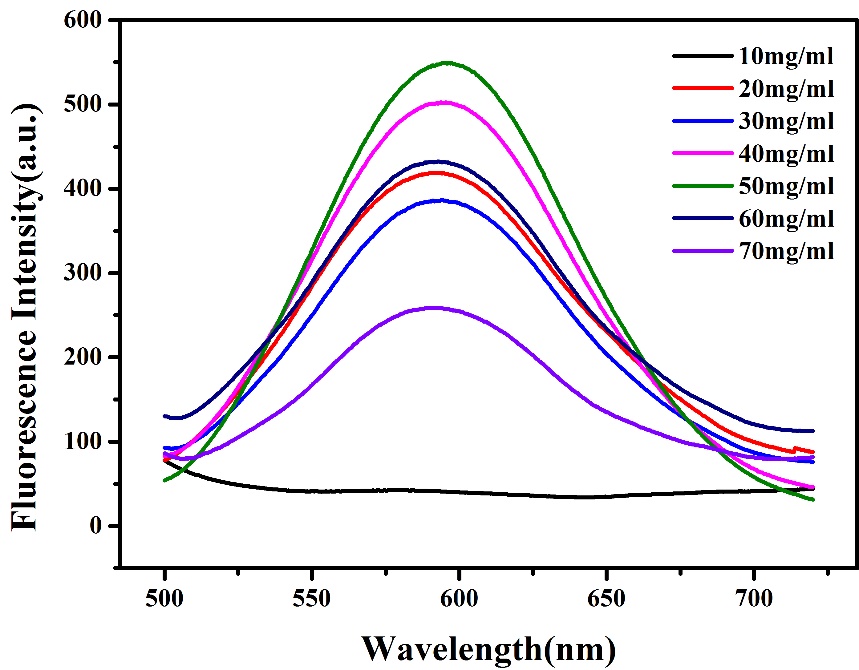


**Figure S3** Fluorescence intensities of Cu nanoclusters when the concentrations of glutathione varying from 10 mg/mL to 70 mg/mL were employed.


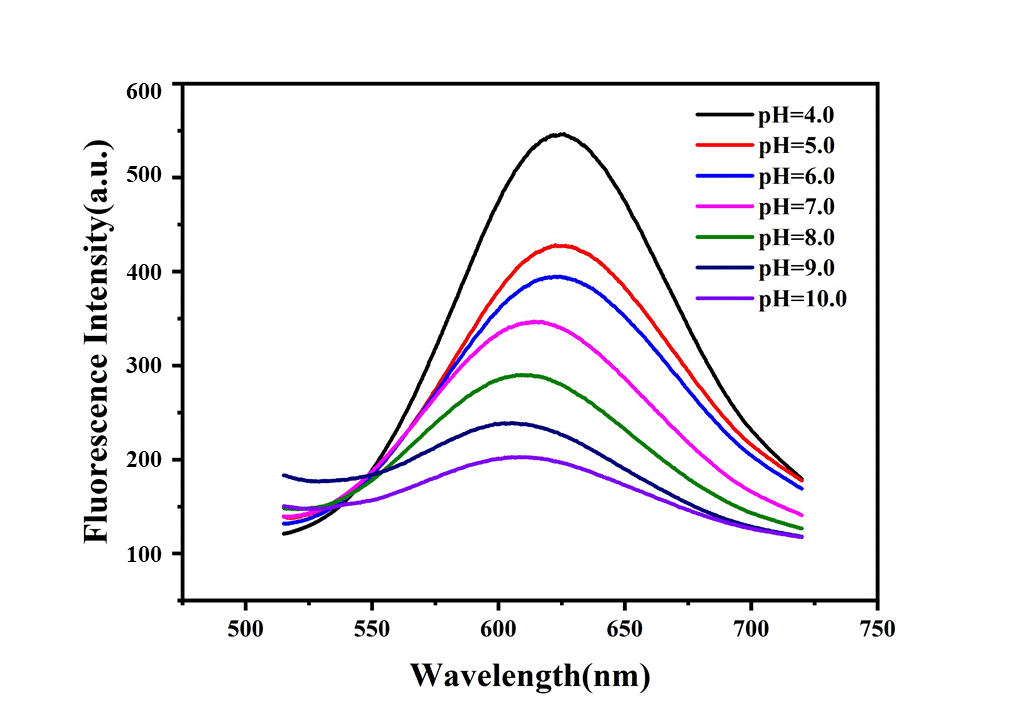


**Figure S4** Fluorescence intensities of Cu nanoclusters when pH values varying from 4 to 10 were employed.


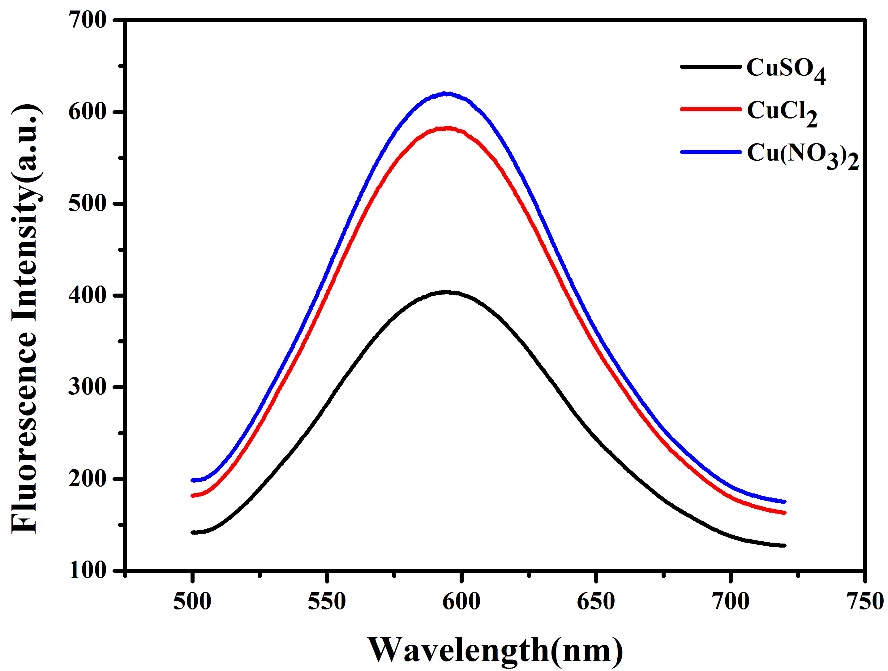


**Figure S5** Fluorescence intensities of Cu nanoclusters when different copper precursors were employed.


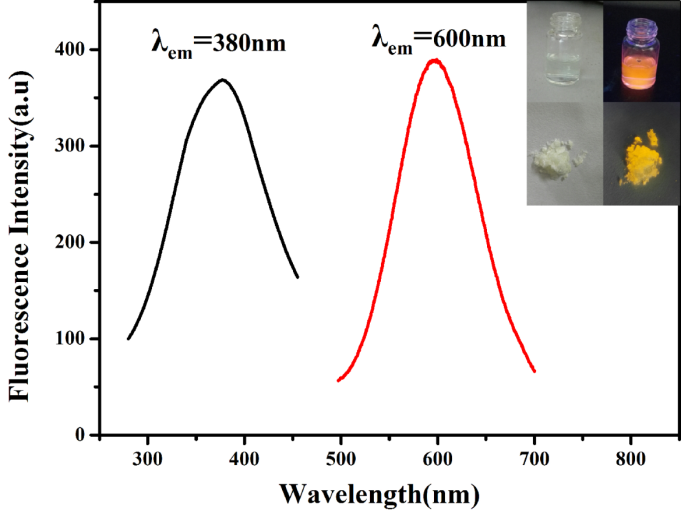


**Figure S6** Fluorescence excitation and emission spectra of Cu nanoclusters. Inset: Images of Cu nanoclusters powder and solution under sunlight and UV light.


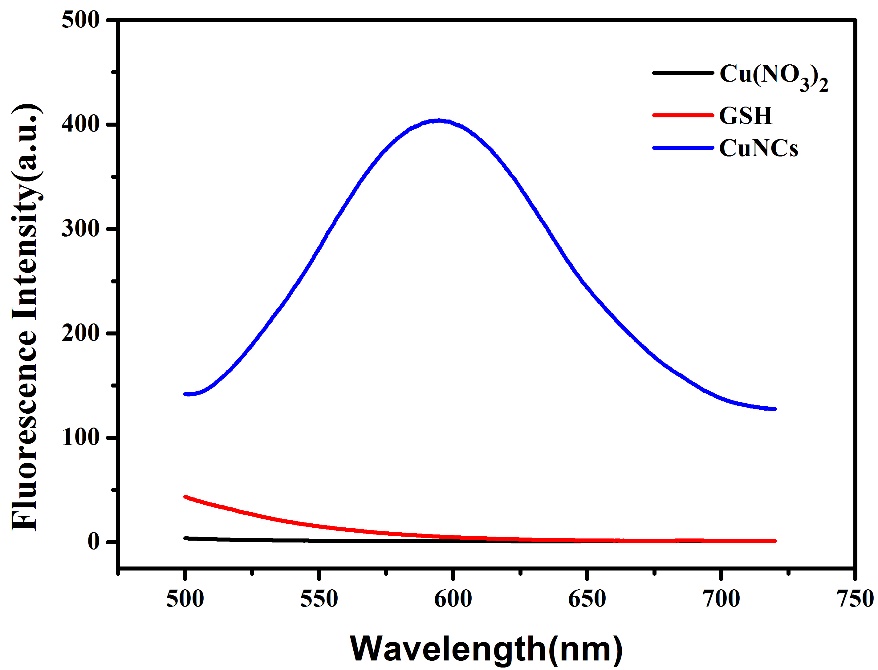


**Figure S7** Fluorescence emission spectra of Cu nanoclusters, glutathione and Cu(NO_3_)_2_.


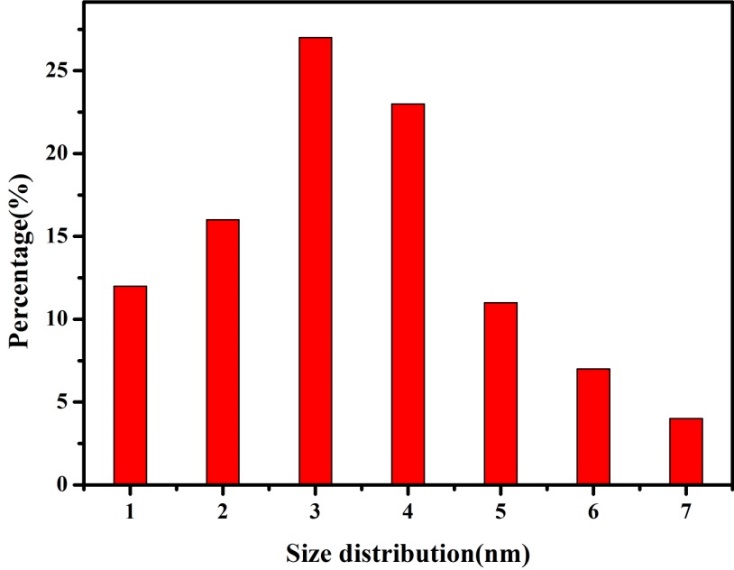


**Figure S8** Size distribution of Cu nanoclusters and the size is about 3 nm.

**Figure S9** Fluorescence spectra of Cu nanoclusters in the presence and absence of As(III). *Inset*: Fluorescence images of Cu nanoclusters in the presence (A) and absence (B) of As(III) under a 365 nm UV light.

**Figure S10** The influence of pH values varying from 4 to 10 on the sensitive detection of As(III) by Cu nanoclusters.


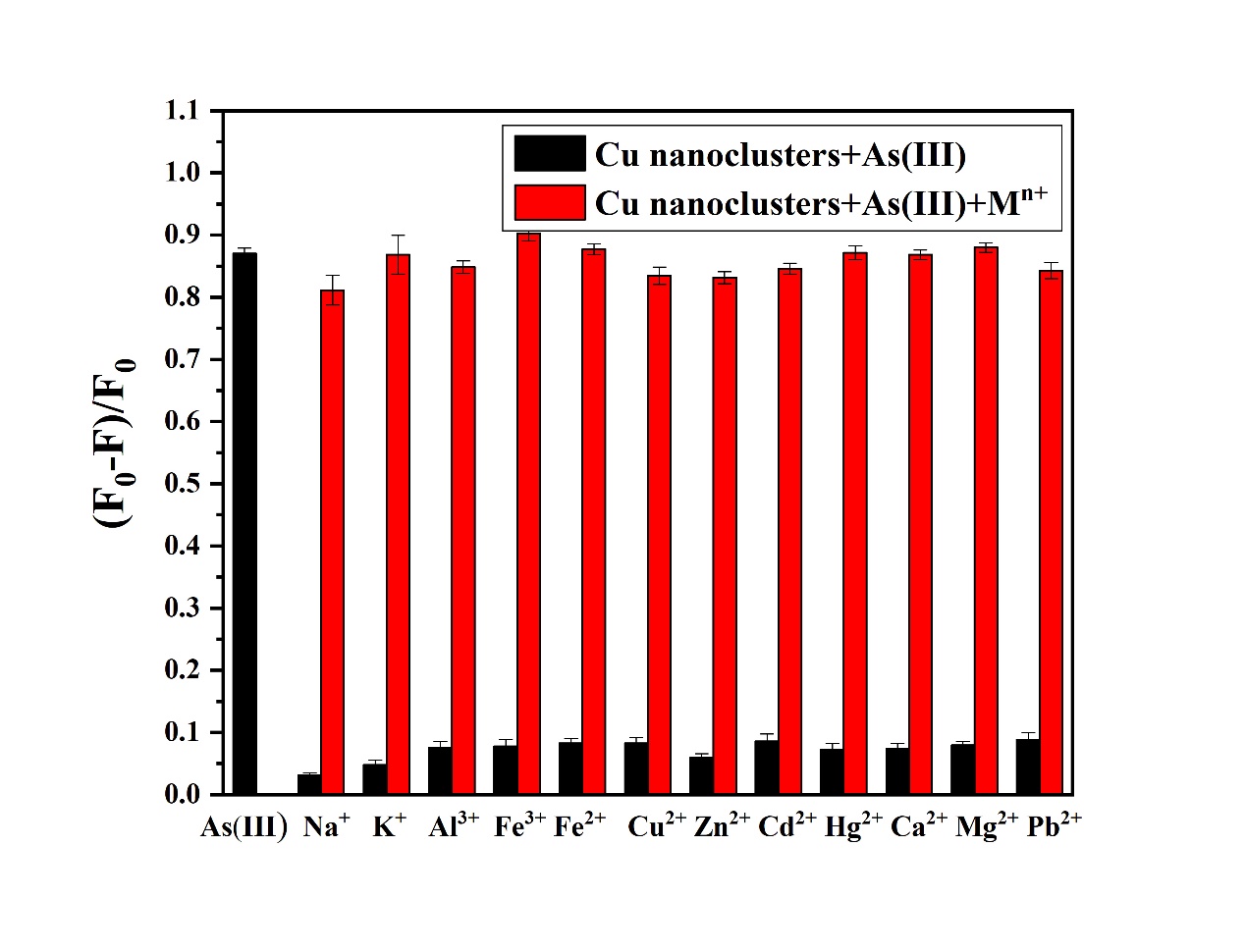


**Figure S11** The selectivity of Cu nanoclusters toward As(III) over common cations. The black bars represent the fluorescence response of Cu nanoclusters toward As(III) (0.5 μM) and other interfering cations (As(III) (10 μM). The red bars represent the fluorescence response Cu nanoclusters toward interfering cations (10 μM) in the presence of 0.5 μM As(III). All experiments were carried out in BR buffer solution.

**Table S1** Comparison of detection limit of Cu nanoclusters with other previously reported sensors for As(III) detection.

| As(III) detection sensors | Detection limit | References |
| --- | --- | --- |
| Gold nanoparticles on cellulose membranes | 13.32nM (10 ppb) | 32 |
| Gold cluster-based fluorescent sensor | 53.67 nM (4.03 ppb) | 33 |
| Cationic polymers and aptamers | 70.59 nM (5.3 ppb) | 34 |
| Fluorescence CdS quantum dots | 932.3 nM (70 ppb) | 35 |
| Potentiometric membrane electrode | 499.4 nM (37.5 ppb) | 36 |
| Alginate immobilized pumpkin urease | 174.7 nM (13.12 ppb) | 37 |
| Aptamer-modified nanogold | 25.30 nM (1.9 ppb) | 38 |
| Gold Nanoparticle-Modified Glassy Carbon Electrodes | 24.78 nM (1.861 ppb) | 39 |
| Hemin peroxidase | 79.91 nM (6 ppb) | 40 |
| Cu nanoclusters | 2.93 nM (0.22 ppb) | This work |

**Table S2** The recovery test of As(III) in ultrapure water, tap water and groundwater samples by Cu nanoclusters ^a^.

| Spiked concentrations/nM | Ultrapure water | | Tap water | | Groundwater | |
| --- | --- | --- | --- | --- | --- | --- |
|  | Found/nM | Recovery/% | Found/nM | Recovery/% | Found/nM | Recovery/% |
| 50 | 50.4±0.8 | 100.8±1.6 | 51.1±0.8 | 102.2±1.6 | 52.1±0.5 | 104.2±1.0 |
| 100 | 99.3±0.3 | 99.3±0.3 | 98.2±0.9 | 98.2±0.9 | 101.2±0.3 | 101.2±0.3 |
| 150 | 151.2±0.7 | 100.8±0.5 | 150.8±0.5 | 100.5±0.3 | 149.8±0.4 | 99.8±0.3 |

^a^ Average±S.D. (*n* = 3).

**Table S3** As(III) concentrations determination from HPLC-ICP-MS and smartphone integrated paper sensors.

| Collected areas of natural groundwater | As(III) concentrations from HPLC-ICP-MS/μM | As(III) concentrations from paper sensors/μM |
| --- | --- | --- |
| Datong basin | 1.09 ± 0.05 | 1.12 ± 0.03 |
| Xiantao | 0.41 ± 0.03 | 0.46 ± 0.02 |
| Foshan | 0.16 ± 0.02 | 0.15 ± 0.03 |
| Kuitun | 0.85 ± 0..07 | 0.87 ± 0.04 |
